# The Development of Lateralized Brain Oscillations in Infants: Lessons From Autism

**DOI:** 10.1002/dev.70101

**Published:** 2025-11-05

**Authors:** Gabriel Blanco‐Gomez, Christian O'Reilly, Sara Jane Webb, Mayada Elsabbagh, The BASIS Team

**Affiliations:** ^1^ Montreal Neurological Institute‐Hospital Montreal Canada; ^2^ McGill University Montreal Canada; ^3^ Artificial Intelligence Institute University of South Carolina Columbia South Carolina USA; ^4^ Department of Computer Science and Engineering University of South Carolina Columbia South Carolina USA; ^5^ Carolina Autism and Neurodevelopment Research Center University of South Carolina Columbia South Carolina USA; ^6^ Institute for Mind and Brain University of South Carolina Columbia South Carolina USA; ^7^ Center on Child Health, Behavior and Development, Seattle Children's Research Institute Seattle Washington USA; ^8^ Centre for Brain and Cognitive Development London UK

**Keywords:** autism spectrum disorder, brain asymmetry, brain development, brain lateralization, electroencephalography, language

## Abstract

The lateralization of brain activity is important for language processing and attention, and atypical patterns of lateralization have been linked to many neurodevelopmental disorders, including autism spectrum disorder (ASD). However, the developmental timing of these patterns and their relationship to emerging ASD characteristics are unclear. In this study, we used data from EEG‐IP (International Infant EEG Data Integration Platform), a longitudinal cohort bringing together infants at elevated likelihood for ASD and age‐equivalent controls across two sites. We examined brain lateralization in electroencephalography (EEG) power during the first year of life. Overall, we identified differences in gamma band lateralization in infants later diagnosed with ASD at 12 months but not at 6 months. Additionally, we observed a shift from high left gamma band asymmetry at 6 months toward more symmetry by 12 months in our control group, highlighting between‐group differences in developmental trajectories in brain oscillatory activity. We found key differences in the lateralization across groups in brain regions within the auditory network, which is thought to be important for language learning. Overall, examining the developmental trajectories of lateralization is a crucial step toward creating more accurate models of brain development and better understanding the underlying mechanisms of neurodevelopmental disorders.

## 1 Introduction

The human brain has been known to have two hemispheres with differing functions for more than a century (Broca 1861; Kimura and Archibald 1974; H. Liu et al. 2009; Oades 1998). Processing of linguistic input activates regions in the left hemisphere (S. Wang et al. 2019), whereas the right hemisphere is associated with other higher order functions, such as attention (Russell‐Giller et al. 2021; Śmigasiewicz et al. 2017) and visuospatial integration (Gotts et al. 2013). The simultaneous and independent processing of information by each hemisphere is considered to be crucial for cognitive capacity (Rogers 2021). This concept aligns well with computational theories of functional specialization, positing that having distinct functions and a division of processing can improve performance for the completion of complex cognitive tasks (Gotts et al. 2013).

In infants, lateralization emerges early during the first trimester of gestation (Corballis 2013), and the development of functional brain asymmetry plays a key role in achieving linguistic potential (Spironelli and Angrilli 2010). Although a handful of landmark studies have been published regarding lateralization in typically developing infants (Anaya et al. 2021; Brooker et al. 2017; Emerson et al. 2016; Gartstein et al. 2020; Howarth et al. 2016), findings have been limited for various reasons. First, these studies have focused exclusively on alpha power (neural activity within the 6–13 Hz range in adults and 6–9 Hz in infants), even though research in adults has shown that lateralization of higher frequency bands can modulate various cognitive processes (Adam et al. 2020; Benasich et al. 2008; Cartocci et al. 2021; Morillon et al. 2012). Second, they primarily focus on frontal activity because of its association with internal behaviors, emotions, and temperament (Brooker et al. 2017), although lateralization can be found in various regions across the brain. Thus, considering the functional and anatomical segregation of brain oscillations and the importance of lateralization during development, a detailed and nuanced account of how brain lateralization develops is needed.

Moreover, there is limited understanding of how brain lateralization patterns differ in neurotypical infants compared to infants with an elevated likelihood (EL) of autism spectrum disorder (ASD). ASD is a neurodevelopmental condition characterized by a wide range of symptoms, causes, and endotypes (American Psychiatric Association 2013). Although a diagnosis of ASD can be made as early as 24 months (Elsabbagh 2020), differences in brain development start much earlier, even as soon as 3 months of age (Wolff et al. 2012). To further understand developmental trajectories, research on early autism biomarkers has focused on studying younger siblings of children with ASD. Familial recurrence rates for ASD are approximately 20% (Ozonoff et al. 2024), and many of these children exhibit some autism symptoms compared to the general population (Messinger et al. 2013). Thus, studying patterns of brain development in this population can provide crucial insights into the early markers of ASD.

Previous studies using electroencephalography (EEG) in infants with EL for ASD have revealed important differences during the first year of life (Cantiani et al. 2019; Carter Leno et al. 2021; Finch et al. 2017; L. Gabard‐Durnam et al. 2015; L. J. Gabard‐Durnam et al. 2019; Hazlett et al. 2017; Huberty et al. 2021, 2023; Jones et al. 2020; Keehn et al. 2015; Levin et al. 2017; O'Reilly et al. 2023; Tierney et al. 2012). Importantly, differences in the development of hemisphere specialization are hypothesized to index ASD symptoms (D'Mello et al. 2016; Floris et al. 2016; Li et al. 2023; Wolff et al. 2012). For example, a recent meta‐analysis of neuroimaging studies in autism revealed that differences in structural and functional lateralization were associated with clinical measures of communication, reciprocal interactions, and repetitive behaviors (Li et al. 2023). Similarly, increased functional lateralization of motor circuits has been associated with lower performance in motor tasks in children with ASD (Floris et al. 2016). Brain lateralization encompasses the balance between left and right brain activation, and disruptions in this balance can lead to cascading effects across various cognitive domains (Duboc et al. 2015). However, how ASD impacts the development of brain lateralization is still understudied. A few studies have suggested that autistic individuals have more brain lateralization (Eyler et al. 2012; L. Gabard‐Durnam et al. 2015; Keehn et al. 2015), whereas others have found less lateralization (Rolison et al. 2022; Seery et al. 2013) compared to comparison groups. This inconsistency in lateralization studies can be attributed to variations in task design, neuroimaging methods, regions of interest, and age, as well as a lack of normative studies outlining how lateralization emerges in typical development. Taken together, available data on the early developmental period when lateralization patterns emerge are limited (Emerson et al. 2016; J. Liu et al. 2019).

In addition, some of the limitations in this field stem from studies using EEG scalp recordings. EEG signals at the scalp level contain ambiguous information on the origin of brain activity. It is a common misconception that EEG activity measured by scalp electrodes is generated by activity from a brain region directly underneath. Although electrodes measure electrical potential on the scalp, they do not represent activity from localized brain regions. For example, in a study looking at the sources of covert attention in infants, the authors showed that an often replicated ERP component found around the midline was generated by two distinct brain regions within the prefrontal and cingulate cortex (Richards 2005). To address this fundamental limitation, researchers have used various techniques commonly referred to as source localization. These approaches use standard tissue conductivities and information derived from magnetic resonance imaging (MRI), such as skull thickness and anatomical boundaries, in conjunction with high‐density EEG to localize the anatomical sources of neural activity (Michel and Brunet 2019; O'Reilly et al. 2021; Richards 2005). These techniques allow researchers to examine patterns of brain activity across the cortex (or the whole brain) and better capture developmental changes. In the infant literature, these techniques have been successfully used to identify the anatomical sources for cover attention (Richards 2005, 2013) and auditory processing (Cantiani et al. 2019; Hämäläinen et al. 2019; Musacchia et al. 2015), among others. For example, Hämäläinen et al. (2019) showed that infants undergo developmental changes in source strength during an auditory task, a finding only possible using source reconstruction. Similarly, in an infant EEG study, Thorpe et al. (2016) used source localization analyses to show that alpha desynchronization originates mostly from central and parietal regions at 12 months of age and changes toward more frontal regions later in development.

The current study aims to take advantage of these methodological approaches to explore the development of EEG power lateralization across a wide range of frequencies. Using a sibling study design, we will examine the emergence of lateralization of neural oscillations in the brain during the first year of life, comparing typically developing infants and those at EL for ASD. Moreover, we will employ mixed‐effect linear models to examine how these developmental patterns are modulated by EEG frequency bands (theta, alpha, beta, and gamma) across distinct brain networks.

On the basis of the current infant literature, we hypothesize that there will be differences in both the direction and magnitude of lateralization across frequency bands. Specifically for the alpha band, a study comparing 6‐month‐olds at EL for ASD and neurotypical controls found that infants at EL for autism displayed higher left‐hemisphere lateralization (L. Gabard‐Durnam et al. 2015), although other studies have reported no hemisphere differences in connectivity (Orekhova et al. 2014). For the theta band, a sibling study identified differences in left‐hemisphere theta activity in infants at EL for autism compared to controls (Piazza et al. 2023), though lateralization effects were not directly examined. In the gamma band, increases in left‐hemisphere activity have been reported in boys with autism relative to age‐matched controls, suggesting that differences in gamma lateralization may emerge earlier in development. Beta band oscillations, however, remain relatively understudied. In a recent systematic review, the authors reported consistent evidence for differences in resting‐state gamma and alpha power, but only limited evidence for theta and beta differences (Neo et al. 2023). Given the current evidence, we hypothesize that infants in our sample will display left‐hemisphere dominance in alpha and gamma bands, more subtle differences in the theta band, and limited differences in beta band.

Another aim of this study is to identify the EEG sources of global lateralization in the brain and assess how they differ across diagnostic groups. Although infant studies have widely discussed brain lateralization over frontal regions (Finch et al. 2017; Kühn‐Popp et al. 2016; Seery et al. 2013), conclusions regarding the lateralization of specific EEG sources remain limited. During tasks that involve auditory stimuli, source localization in infants has identified hemispheric differences in the auditory cortex (Cantiani et al. 2019) as well as parts of the mid‐cingulate cortex (Piazza et al. 2016). Similarly, fMRI analyses have also observed lateralization differences at 9 months of age in the posterior cingulate cortex (Rolison et al. 2022) in typical populations. Therefore, we hypothesize that our source localization approach will identify regions of the auditory cortex and cingulate cortex as being highly lateralized. Further, we expect to find stronger left hemisphere dominance in infants at higher risk for autism compared to their typically developing counterparts, consistent with scalp‐level findings (L. Gabard‐Durnam et al. 2015). Overall, this study seeks to understand the development of lateralized brain oscillations. By using source reconstruction and taking advantage of this study's longitudinal design, we aim to shed light on how lateralization emerges in typical development and in ASD.

## 2 Materials and Methods

### 2.1 Participants

This study relied on data from the International Infant EEG Data Integration Platform (EEG‐IP), a multi‐site pooling of two longitudinal cohorts of infants at EL for autism (van Noordt et al. 2020). Participants in this study were assigned to one of two groups: infants at EL of autism, defined as those with familial risk by virtue of having a sibling with an ASD diagnosis, and the comparison group, defined as infants with no known family history of autism and, therefore, having a typical likelihood for ASD (TL). Data were collected at two sites as part of independent projects: Birkbeck, University of London in the United Kingdom (EEG collected around 7 and 14 months) and the University of Washington‐Seattle in the United States (EEG collected around 6, 12, and 18 months). A detailed account of the longitudinal breakdown can be found in Table S1.

In both sites, clinical assessments were conducted at 24 and 36 months. Diagnoses were ascertained by clinicians and informed, among other criteria, by scores on the Autism Diagnostic Observation Schedule‐Generic (ADOS‐G). All ADOS scores were calibrated and standardized (Hus and Lord 2014). The original study consisted of EEG recordings from 195 infants, of whom 10 participants were excluded because they did not attempt the video‐watching paradigm. An additional 10 participants were excluded due to insufficient EEG epochs after artifact rejection (see the following section for details). As a result, data from 175 participants were analyzed (London *N* = 96; Washington *N* = 76), which includes 140 EEG recordings at 6–7 months (hereafter, referred to as the 6‐month time point) and 152 EEG recordings at 12–14 months (hereafter, referred to as the 12‐month time point). A total of 117 participants had recordings at both time points. Moreover, 30 infants were later diagnosed with ASD, including three participants from the TL group. These three participants were included in the ASD versus non‐ASD group and excluded from the TL versus EL analyses. Participant statistics and demographics after data exclusions can be found in Table 1.

**TABLE 1:** Participant demographics.

Age	Group	Outcome	*N* (females)	Total
6–7 months	EL	ASD	19 (8)	69
		Non‐ASD	50 (23)	
	TL	ASD	3 (1)	71
		Non‐ASD	68 (35)	
	Combined	ASD	22 (9)	140
		Non‐ASD	118 (58)	
12–14 months	EL	ASD	24 (11)	79
		Non‐ASD	55 (27)	
	TL	ASD	2 (1)	73
		Non‐ASD	71 (39)	
	Combined	ASD	26 (12)	152
		Non‐ASD	126 (66)	

*Note:* Participant demographics by age, likelihood group, and diagnostic outcome.

Abbreviations: ASD, autism spectrum disorder; EL, elevated likelihood; TL, typical likelihood.

### 2.2 EEG Data Acquisition

High‐density EEG was recorded while participants watched 30–40 s videos, presented in a randomized order. These videos included (1) a woman singing nursery rhymes, (2) brightly colored toys moving, and (3) brightly colored toys manipulated by a human hand (London only). EEG data were acquired using a 128‐channel HydroCel Geodesic Sensor Net and the Net Station software (Electrical Geodesics, Eugene, Oregon). To remove power line contamination, a notch filter was applied to the London (50 Hz) and Seattle (60 Hz) recordings. EEG was recorded at 500 Hz with a vertex (Cz) reference and re‐referenced to an average reference.

Recordings were standardized and preprocessed with the EEG‐IP‐Lossless pipeline (Desjardins et al. 2021). Noisy channels and epochs were identified and removed. Independent components associated with non‐neural sources (e.g., EOG components) were rejected. Annotations for noisy channels and epochs and the independent component classification were reviewed by an expert to confirm automated classification (Desjardins et al. 2021). Preliminary analyses revealed no significant differences in spectral EEG power between each of the three video conditions (*p* > 0.05). Therefore, recordings from all videos were pooled together, segmented into 1‐s non‐overlapping epochs, and considered representative of a resting state. The use of a video‐watching paradigm to collect resting‐state data is customary in infants, as the use of videos increases the chances of obtaining recordings in a quiet, restful state in this population. The epochs were used for source reconstruction and calculating lateralization indices. The mean number of epochs did not differ between groups (*p* > 0.05). We excluded six participants because they had less than 20 epochs, an insufficient number for adequate analysis (O'Reilly et al. 2023). In addition, we excluded all participants whose EEG spectral power (SP) values were ±3 standard deviations away from the mean for individual EEG frequencies. This threshold is consistent with established practice for artifact rejection and corresponds to rejecting values more extreme than 99.7% of the Gaussian distribution (Miskovic et al. 2009).

Activity from all electrodes was used to identify sources, given the focus on identifying whole‐brain patterns during development. Source reconstruction was estimated using age‐appropriate head templates (O'Reilly et al. 2021). These templates were built from MRI averages using the boundary element method (BEM) segmentation of head tissue. Because digitized electrode positions were not available, electrode locations were assigned using the HydroCel GSN 128‐channel positions as defined in Richards et al. (2015). We estimated all sources using the 12‐month template to avoid systematically confounding the effect of using different templates with the age at the time of EEG recording. Sources were estimated using the MNE Python package (Gramfort et al. 2013) and the eLORETA inverse operator (Pascual‐Marqui et al. 1994), with *λ* ^2^ = 10^−4^. A total of 66 regions were obtained from the original templates based on the Desikan–Killiany parcellation (Desikan et al. 2006), which relies on Infant FreeSurfer (Zöllei et al. 2020). This parcellation is based on the M‐CRIB atlas (Alexander et al. 2017), an effort to replicate the Desikan parcellation in infants to enable consistency between adult and infant brain region nomenclature. Sources were averaged within each brain region. All regions extracted are available in Table S2.

### 2.3 EEG Lateralization Index (LI)

Lateralization was derived from the absolute SP. SP was computed from the standardized EEG‐IP recordings using MNE‐Python (Gramfort et al. 2013). For each recording, the power spectrum was averaged across epochs. An LI was then calculated using the formula LI = (LH − RH)/(LH + RH), where LH and RH are the left‐hemisphere and right‐hemisphere SP, respectively, for homotopic brain regions (Thut 2006). LI values were calculated for the following frequency bands: theta 4–6 Hz, alpha 6–13 Hz, beta 13–30 Hz, gamma 30–50 Hz, broadband 4–50 Hz. Values for each frequency band were calculated by averaging the SP within each individual range (e.g., 6–13 Hz for the alpha band).

### 2.4 Statistical Analysis

To characterize how lateralization arises in typical neurodevelopment, we conducted a two‐way repeated measures ANOVA using age (6, 12 months) and frequency band (theta, alpha, beta, gamma) as within‐subjects factors. This analysis was limited to infants in the TL group. Second, we ran a three‐way mixed effects ANOVA using age (6, 12 months) and frequency band (theta, alpha, beta, gamma) as within‐subjects factors and likelihood (EL, TL) as between‐subject factors. A third analysis was carried out using diagnostic outcomes (ASD vs. non‐ASD) as between‐subject factors. Greenhouse–Geisser sphericity corrections were applied to within‐subject factors that violated the sphericity assumption. We further examined significant main effects and interactions with paired‐sample *t*‐tests. Bonferroni corrections were applied to correct for multiple tests. Throughout the analyses, we kept a strict *α* = 0.05, although effects that were marginally significant (0.05 < *p* < 0.10) are also reported. These analyses were done using the “rstatix” package in R (v0.7, Kassambara 2021).

A mixed‐effects multi‐factorial linear regression model was also used to assess the effect of the likelihood of ASD, age, and biological sex on lateralization (fixed effects). Nested random effects were included for each subject. Two models were included in this study:

1. LI ∼ likelihood × age + sex + site
2. LI ∼ outcome × age + sex + site

Models (1) and (2) were used to test the interaction of age and ASD likelihood and ASD diagnosis, respectively, on brain lateralization. Statistical analyses were run using various Python packages, mainly statsmodels 0.9.0 for linear regression, pandas 1.4.0 for data manipulation, and seaborn 0.11.0 for visualization. Finally, to assess the developmental trajectories for different brain networks, we selected bands with significant interactions and calculated the same mixed effects model for three core brain networks. These include the visual, auditory, and default mode networks, as defined by a previous study on infants (S. Wang et al. 2021). Laterality values for each network were obtained by averaging all lateralization values for homotopic regions within a network. In addition, given the extensive research supporting the lateralization of language areas, we also calculated the mean lateralization scores for regions within the language network as defined by Shirer et al. (2012).

## 3 Results

### 3.1 Lateralization Decreases Across the First Year of Life

We first examined how lateralization changes during development in the EL and TL groups. A repeated‐measures ANOVA showed a statistically significant main effect for age (*F*_1,155_ = 6.16, *p* = 0.011) but no effects of frequency bands. Follow‐up analyses showed an overall significant decrease during the first year of life (*p* < 0.001). This effect was characterized by a shift from strong left‐hemisphere lateralization at 6 months toward no lateralization at 12 months. The effect of age was also significant in alpha (*p* < 0.01) and theta (*p* < 0.01) but not in the other frequency bands.

**FIGURE 1:**
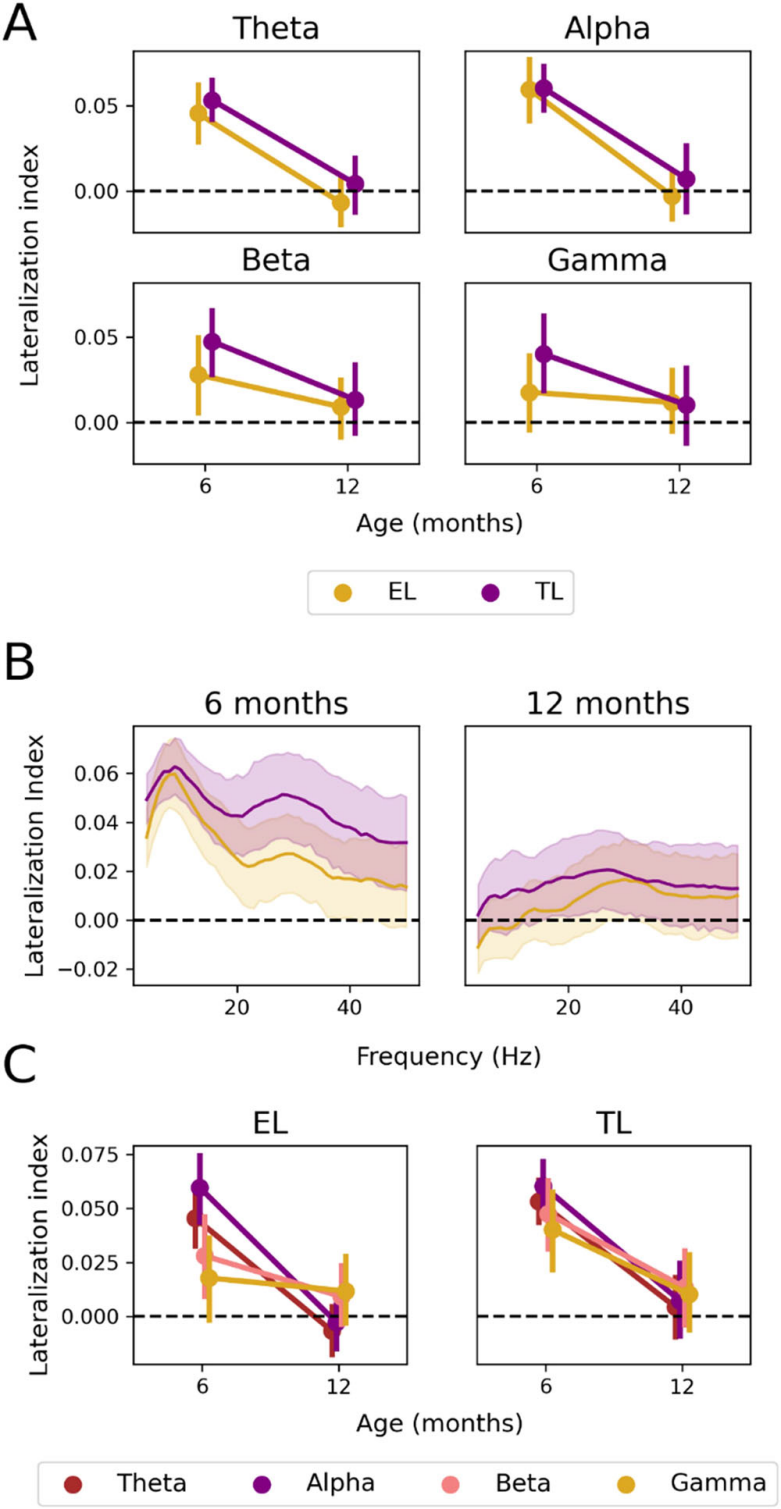
(A) Mean lateralization indices as a function of age (6 and 12 months) for each frequency band: theta, alpha, beta, and gamma. Contrasts between likelihood groups: EL (purple), TL (yellow). Negative values represent right‐hemisphere dominance, whereas positive values represent left‐hemisphere dominance. (B) Mean lateralization indices across the EEG frequency spectrum. Cross‐sectional analyses for each time point: 6 (left) and 12 months (right). (C) Changes in mean lateralization for each likelihood group: EL (left) and TL (right). Analyses are presented for each frequency band: theta, alpha, beta, and gamma. EEG, electroencephalography; EL, elevated likelihood; TL, typical likelihood.

We then examined the relationship between the likelihood of ASD and the development of lateralization. No significant effects were found between the ASD likelihood and lateralization at 6 or 12 months. There was only a significant effect of age across groups. Post hoc analyses revealed a significant decrease in alpha and theta lateralization for TL and EL groups (*p* < 0.01). We also found no significant effect of the ASD likelihood on the lateralization in each of the four frequency bands (see Figure 1). Lateralization values for each group and time point can be found in Table 2.

**TABLE 2:** Mean lateralization values per age group.

	Mean lateralization at 6 months (sd)				
*Group*	*n*	**Theta**	**Alpha**	**Beta**	**Gamma**
TL	71	**0.053 (0.050)**	**0.060 (0.058)**	**0.048 (0.082)**	**0.040 (0.095)**
EL	69	**0.046 (0.072)**	**0.060 (0.078)**	**0.028 (0.090)**	0.018 (0.093)
Non‐ASD	118	**0.049 (0.061)**	**0.061 (0.068)**	**0.041 (0.086)**	**0.032 (0.094)**
ASD	22	**0.051 (0.068)**	**0.053 (0.070)**	0.020 (0.090)	0.012 (0.093)
	Mean lateralization at 12 months (sd)				
*Group*	*n*	**Theta**	**Alpha**	**Beta**	**Gamma**
TL	73	0.004 (0.070)	0.007 (0.085)	0.013 (0.087)	0.010 (0.093)
EL	79	−0.007 (0.058)	−0.003 (0.065)	0.009 (0.077)	0.012 (0.087)
Non‐ASD	126	0.003 (0.065)	0.006 (0.077)	0.006 (0.079)	0.003 (0.085)
ASD	26	−0.022 (0.057)	−0.018 (0.062)	**0.038 (0.091)**	**0.050 (0.102)**

*Note:* Mean lateralization values and standard deviations for each age group (6 or 12 months). Items bolded indicate values that are significantly different from zero at *α* = 0.05.

Abbreviations: ASD, autism spectrum disorder; EL, elevated likelihood; TL, typical likelihood.

### 3.2 Lateralization Differences by ASD Outcomes

**FIGURE 2:**
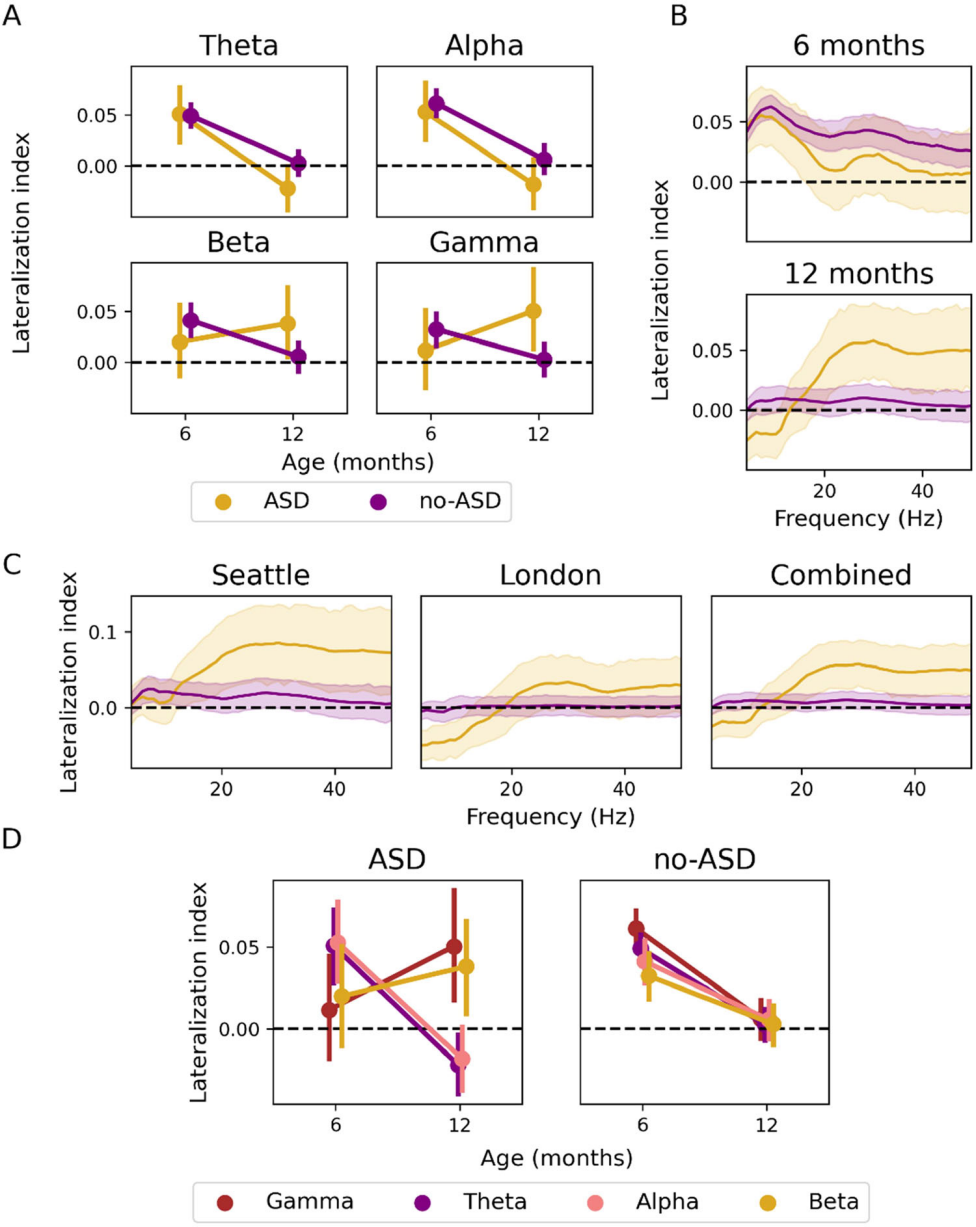
(A) Mean lateralization indices as a function of age (6 and 12 months) for each frequency band: theta, alpha, beta, and gamma. Contrasts between outcome groups: ASD (yellow) versus non‐ASD (purple). Negative values represent right‐hemisphere dominance, whereas positive values represent left‐hemisphere dominance. (B) Mean lateralization indices across EEG frequencies. Cross‐sectional analyses for each time point: 6 months (top) and 12 months (bottom). The ASD group shows right hemisphere lateralization at 12 months of age, whereas the non‐ASD group shows a lack of lateralization. The shaded areas show the 90% confidence intervals. (C) Site differences, across frequencies, at 12 months of age. Analyses are presented for each of the testing sites: Seattle (left), London (middle), and both sites combined (right). (D) Changes in mean lateralization for each outcome group: ASD (left) and non‐ASD (right). Analyses are presented for each frequency band: theta, alpha, beta, and gamma. All changes in lateralization between 6 and 12 months of age are significant, except for changes in gamma lateralization. ASD, autism spectrum disorder; EEG, electroencephalography.

Overall, our analyses revealed a statistically significant three‐way interaction between outcome group (ASD vs. non‐ASD), age, and frequency band (*F*_2,135_ = 9.46, *p* = 0.001). Further inspection revealed a significant group difference only in the gamma band at 12 months (*F*_1,152_ = 6.25, *p* = 0.014), as shown in Figure 2, but not in the other bands (A and B). This points to the fact that the ASD group has a higher gamma lateralization value (more leftward dominance) than the non‐ASD group. Follow‐up analyses for each group revealed that changes in the gamma band lateralization between 6 and 12 months were statistically significant in the non‐ASD group (*p* = 0.01) but not in the ASD group. As such, infants who did not develop ASD saw a shift from left hemisphere dominant lateralization toward no lateralization (Figure 2A). Although we observe a similar trend in beta band activity, these group differences were not statistically significant at 12 months of age (*p* > 0.05). Post hoc analyses also revealed that the shift toward more leftward lateralization was not driven by a significant change within the left or the right hemisphere, indicating that this result is specific to the relation between the two hemispheres (Figure S2). Additional analyses regarding group differences between EL infants who do not develop ASD (EL‐non‐ASD) and TL infants revealed no significant differences (Figure S1). However, EL‐non‐ASD infants had less lateralization than TL and EL‐ASD infants (see Supporting Information). In addition, there were no significant effects or interactions between sex as assigned at birth and changes in lateralization.

### 3.3 Lateralization Across Brain Regions

**FIGURE 3:**
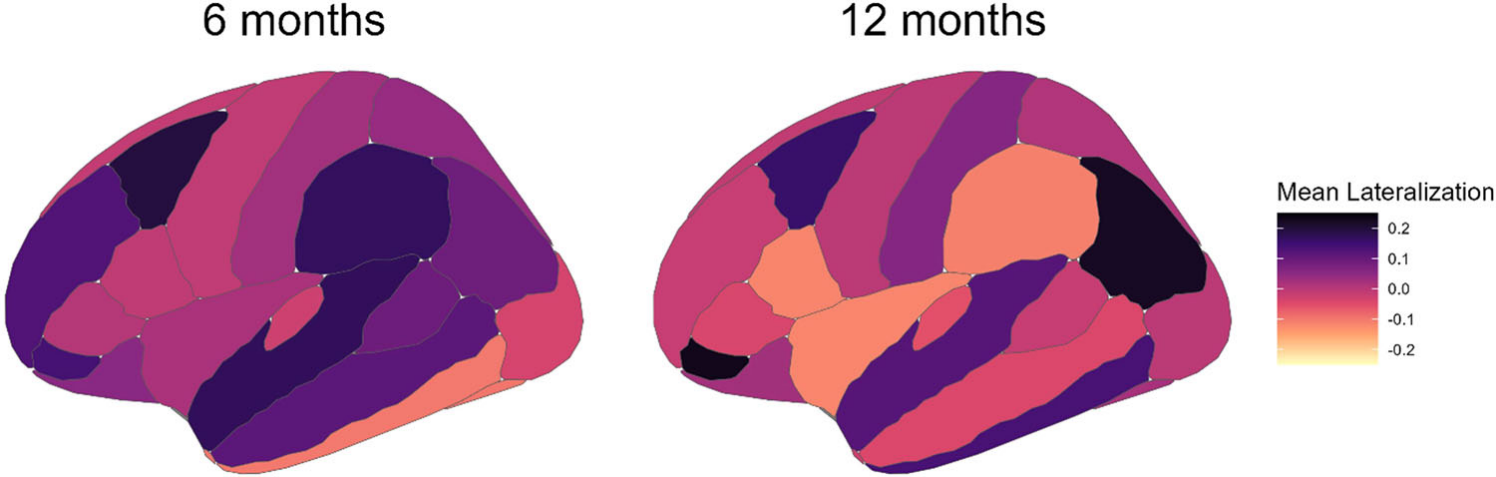
Mean lateralization indices for each brain region based on the DK Atlas parcellation. The image on the left represents lateralization at 6 months, whereas the image on the right represents lateralization at 12 months. Darker colors represent left‐hemisphere dominance, whereas lighter colors represent right‐hemisphere dominance.

Another goal of this study was to examine how lateralization changes in brain networks during typical development. Therefore, we concentrated on the TL group for this analysis. We calculated lateralization scores for 33 homotopic regions (Fillmore et al. 2015) and averaged them across all participants at 6 and 12 months. *Z*‐scores were then calculated for each region, and sources were defined as “lateralized” if their absolute LI score was greater than 0.2, as outlined in previous studies (Agcaoglu et al. 2015). At 6 months of age, the posterior middle frontal gyrus (pMFG) and the superior temporal gyrus (STG) were above this threshold. At 12 months of age, activity generated by the medial orbitofrontal gyrus (mOFG), the inferior parietal lobule (IPL), and the inferior frontal gyrus (IFG) was lateralized. The top regions for each age group are presented in Table 3 and Figure 3. A full list of regions, lateralization values, and their distributions can be found in Table S3 and Figure S3.

**TABLE 3:** Top lateralized sources.

Top lateralized sources at 6 months		
Region	Absolute lateralization index	Dominant hemisphere
Posterior middle frontal gyrus	0.23	Left
Superior temporal gyrus	0.22	Left
Top lateralized sources at 12 months		
Region	Absolute lateralization index	Dominant hemisphere
Medial orbitofrontal gyrus	0.28	Right
Inferior frontal gyrus (pars orbitalis)	0.23	Left
Inferior parietal lobule	0.22	Left

*Note:* Regions with a lateralization value above 0.2 at each time point are shown. These values represent mean lateralization within the gamma frequency band for the TL group only.

### 3.4 Developmental Trajectories of Lateralized Brain Regions

Moreover, we examined developmental changes in the highly lateralized region outlined in the previous section. Analyses revealed a significant effect of age for the STG (*p* < 0.05), the mOFG (*p* < 0.001), and the IFG (*p* < 0.01). In the mOFG, there was a shift from no lateralization at 6 months toward rightward lateralization at 12 months, but no significant differences between diagnostic groups. In the IFG, there was a significant increase toward more leftward lateralization from 6 to 12 months.

### 3.5 Diagnostic Differences in the Development of Brain Networks

**FIGURE 4:**
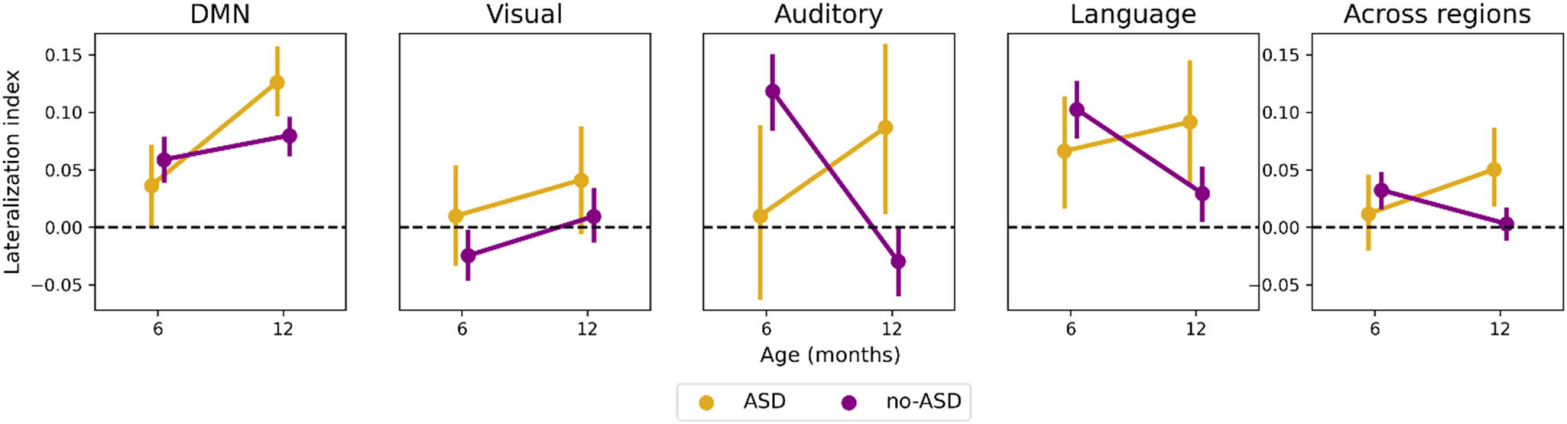
Mean lateralization indices as a function of age (6–12 months). Analyses are presented for each lateralized brain network: default mode network (DMN), visual network, auditory network, language, and across regions. Contrasts between outcome groups: ASD (purple) and non‐ASD (yellow). Negative values represent right‐hemisphere dominance, whereas positive values represent left‐hemisphere dominance. ASD, autism spectrum disorder.

A mixed‐effects multi‐factorial linear regression model revealed a significant interaction effect between age and diagnosis outcome in the auditory network (*p* < 0.001). No significant interactions were found in the default mode, visual, or language networks. Post hoc tests revealed that at 6 months, there was a significant group difference between ASD and non‐ASD in the lateralization scores of the auditory network (*p* = 0.028). On average, the non‐ASD group had more leftward lateralization compared to the ASD group (Figure 4). Moreover, infants in the non‐ASD group saw a significant decrease in lateralization within the auditory network between 6 and 12 months (*p* = 0.04), whereas ASD infants showed a tendency toward an increase. There were also no significant effects for biological sex and testing site. When grouped by ASD likelihood, we found no significant differences in lateralization in any of the brain networks (Figure 4). Additional analyses to examine group differences between EL infants without a positive ASD diagnosis (EL‐non‐ASD) and TL infants also revealed no significant differences (Figure S2).

## 4 Discussion

The aim of this study was to explore the nature and time course of brain lateralization during infancy in typical development and in ASD. Using data from a longitudinal infant cohort, we analyzed resting‐state brain oscillations and revealed four main findings. First, we found that the overall lateralization in the brain decreases in neurotypical infants during the first year of life. More specifically, there was a significant decrease in alpha and theta lateralization. Second, we found that infants later diagnosed with ASD had higher gamma band lateralization at 12 months of age when compared to the TL group. Third, using source estimation, we found four highly lateralized brain regions in infants not diagnosed with ASD. These include the pMFG and STG at 6 months and the mOFG and IFG at 12 months. Although we found no significant effects of ASD likelihood on brain lateralization, brain regions within the auditory network displayed significant differences in lateralization between the ASD and non‐ASD groups.

Our findings with the typically developing group are consistent with recent lateralization studies using diffusion MRI reporting an overall left‐hemisphere bias by 6 months (T. Liu et al. 2021). We additionally noted a shift toward less lateralization at 12 months of age, coinciding with recent studies suggesting that both hemispheres are equally active during the first 2 years of life (Anaya et al. 2021; Emerson et al. 2016). This shift toward symmetry or simultaneous bilateral activation is hypothesized to indicate the early development of interhemispheric cross‐communication and the establishment of complex functional networks. Brain lateralization is an evolutionary adaptation promoting efficient brain processing, cognitive capacity, and information allocation (Rogers 2000, 2021). Our study provides evidence that this adaptation is acquired early during brain development.

Consistent with existing studies, our findings highlight the importance of the gamma band, where we identified differences in lateralization between ASD and non‐ASD infants. Gamma band power has been associated with emotion recognition and face recognition (Yang et al. 2020) as well as perception, selective attention, memory, motivation, and behavioral control (Cartocci et al. 2021; Herrmann et al. 2004; Sirota et al. 2008). In a comprehensive review, Bosman et al. (2014) argued that gamma band activity does not possess a single universal function in the brain but acts as a global coordination system spanning multiple cognitive processes. Gamma band activity is linked to fast‐spiking gamma‐aminobutyric acid (GABA) interneurons thought to drive inhibitory pathways (Bosman et al. 2014; Buzsáki and Wang 2012; Tozzi 2015). In our sample, the increase in left gamma lateralization displayed by the ASD group could signal a disruption of the developmental processes that regulate inhibition in the brain, supporting the excitation–inhibition imbalance theory of autism (Sohal and Rubenstein 2019; Zikopoulos and Barbas 2013). In this theory, many of the phenotypes seen in autism result from increased inhibition or excitation in the brain's signaling pathways. The balance of excitatory and inhibitory mechanisms is crucial for the opening and closure of critical periods, where the infant brain is malleable and undergoes significant changes (LeBlanc and Fagiolini 2011; Sohal and Rubenstein 2019). Therefore, an increase in the lateralization of inhibitory systems can disrupt this balance and result in a wide range of behaviors such as language delays.

One potential cause for these differences in lateralization can be linked to genetic influences. Although differences in gamma band power have been reported in autism during childhood (C.‐G. Wang et al. 2022) and adolescence (Laugeson et al. 2012), our findings indicate that these patterns arise during the first year of life, significantly earlier than previously described. Structural and functional lateralization of the brain are thought to begin during fetal development due to gene‐driven changes (França et al. 2024; Karolis et al. 2023). This is especially relevant in the case of ASD given that a significant overlap exists between genes associated with autism and genes thought to be necessary for hemisphere lateralization (Postema et al. 2019).

A secondary goal of this study was to examine whether ASD likelihood was associated with differences in lateralization patterns. We hypothesized that infants in the EL group would show more lateralization than the TL group. However, we found no significant interactions between ASD likelihood and lateralization. This is consistent with previous studies on lateralization and cortical thickness (Finch et al. 2017; Hazlett et al. 2017), where ASD likelihood was not enough to drive changes in lateralization. Moreover, we found no evidence of sex effects on lateralization. Despite research suggesting that males have more brain asymmetry than females (Kovalev et al. 2003), male and female participants exhibited similar trajectories in lateralization. We also found no significant interactions between site and the diagnostic group factors. Autistic infants had nearly identical patterns of lateralization at 12 months in both London and Seattle, further strengthening the validity of our results and supporting previous claims that the EEG‐IP‐Lossless pipeline can successfully harmonize data across sites (Desjardins et al. 2021). Considering the discrepancies that exist in EEG studies in infants, replicating findings across independent sites is promising.

Our findings also highlight unique lateralization patterns in various brain networks. Using source localization techniques, we were able to document the lateralization of brain oscillations in infants, offering new avenues to document hemisphere asymmetries in clinical and non‐clinical populations. Using age‐appropriate templates, we found five highly lateralized regions during the first year of life, including the pMFG, mOFG, IFG, IPL, and STG. Across all groups, we found a significant increase in the IFG toward more left‐hemisphere dominance between 6 and 12 months of age. Our findings add to the long list of studies reporting a left‐hemisphere dominant lateralization of the IFG and its role in language (Broca 1861; Foundas et al. 1998; S. Wang et al. 2019). This functional and structural lateralization of the IFG has also been shown to develop in infancy, as studies have shown lateralization when exposed to various speech stimuli (Olulade et al. 2020). Although these findings were observed using fMRI, several studies have shown a correlation between fMRI BOLD responses and EEG gamma oscillations (Leicht et al. 2022; Murta et al. 2017; Scheeringa et al. 2016), corroborating the early lateralization of gamma in the IFG. More recently, a study on neonates where EEG *source‐derived* measures of network properties (connectivity, modularity, etc.) showed robust correlations with fMRI connectivity, whereas *scalp‐derived* calculations produced weaker correlations (Chirumamilla et al. 2023). We also saw a significant shift from symmetry at 6 months toward right hemisphere dominance at 12 months for the mOFG. The orbitofrontal cortex has been previously associated with various functions, including mood and reward stimulus association (Campbell‐Meiklejohn et al. 2012; Nitschke et al. 2004). It has also been shown to activate during gentle touch in infants (Kida and Shinohara 2013). However, lateralization patterns of the mOFG and their role in sensory processing are not well understood. These findings further support the use of source localization, which has been previously shown to improve our understanding of brain development in infant populations compared to scalp‐level analyses (Chirumamilla et al. 2023; Richards 2013; Thorpe et al. 2016).

Finally, in terms of brain networks, only the auditory network revealed differences between the ASD group and the non‐ASD group. The auditory network, comprised in large part by the posterior part of the STG and the insular cortex, is associated with phoneme discrimination and auditory attention (Luo and Poeppel 2007; Morillon and Baillet 2017). The auditory network is also one of the earliest identifiable brain networks in neonates (Thomason et al. 2013) and one of the primary “cortical hubs” in the developing brain (Fransson et al. 2011). Research has shown that this network is also highly lateralized, and language deficits have been theorized to be caused by a failure of the left hemisphere to specialize (Eyler et al. 2012). In our sample, neurotypical infants had strong lateralization values at 6 months and then shifted toward less lateralization by 12 months. The opposite is true for infants later diagnosed with ASD, as they start with a lateralization value near zero (symmetry) at 6 months and then lateralize toward the left hemisphere by 12 months. We hypothesize these differences could be due to synaptic pruning. There is evidence of synaptic dysfunction in ASD, which results in aggressive over‐pruning of axons (Hansel 2019; Thomas et al. 2016). These synaptopathies can lead to over‐activation of one hemisphere and differing lateralization patterns, leading to a wide array of symptoms. Given that gamma band activity in the right hemisphere is associated with sensory integration of auditory and linguistic input, higher activation of the right hemisphere could signal deficiencies in sensory integration and language processing. Similarly, Rolison et al. (2022) found that children with ASD had stronger intrahemispheric connectivity in the right auditory cortex and less interhemispheric connectivity. Together, these findings suggest that lateralization in autism may be driven by brain regions within the right auditory network having fewer connections with other homologous areas. One of these areas could be the STG, a region that has been hypothesized to play a key role in speech, emotion response, and social cognition (Bigler et al. 2007; Meekings and Scott 2021; Morningstar et al. 2020). Recent evidence highlights the role of the posterior part of the STG in the integration of information from sensory processing areas and limbic regions, acting as a key player in social interactions (H. Wang et al. 2022). In autism, where social communication deficits are prevalent, STG dysfunctions have been linked to social deficits. A study of 45 school‐age children with autism found that hypoactivation of the left STG at rest was correlated with symptom severity (Gendry Meresse et al. 2005). More research is needed to assess how these regions develop during infancy and to what extent differences in lateralization contribute to autism symptoms.

### 4.1 Limitations

We should begin by outlining the limitations of conducting imaging research on infants. Most EEG acquisition techniques have been designed to study adults, and collecting data from infants poses various issues. These include high inter‐individual heterogeneity in infant populations, shifts in established EEG rhythms, failure to locate optimal reference electrodes, poor fit of EEG caps due to varying head sizes, differences in arousal states, and more (Noreika et al. 2020). Understanding these challenges is important, as they could impact the reliability of the EEG recordings in this study. Moreover, we should highlight the decision to group participants of varying age groups. As part of our study design, we grouped participants between 6 and 7 months in the first time point and those between 12 and 14 months in the second time point. Because the brain undergoes major changes in small time windows during infancy, grouping these datasets may lead to a blurring of age‐related findings. Stroganova et al. (2007) argue that contradictions among EEG studies on autism can be due to averaging across age groups during critical developmental periods. These factors should be taken into consideration when interpreting age‐related changes in lateralization.

In addition, the accuracy and precision of source estimation analyses remain a heavily research topic in the EEG literature (Michel and Brunet 2019). This is specifically difficult with pediatric populations because averaged structural models of the head show lower contrast between white and gray matter and have smaller scaled regions of interest (Phan et al. 2018). Various approaches have been developed to address these issues, including an infant version of the FreeSurfer pipeline for the segmentation and surface extraction (Zöllei et al. 2020) and the creation of population‐average templates for infants (O'Reilly et al. 2021). In this study, we used population templates for source reconstructions. However, using MRI templates derived from each participant could greatly improve source reconstruction estimates. We also calculated an LI that compares the EEG sources of one hemisphere relative to the other hemisphere. Because this index is a relative measure of lateralization, it minimizes the impact of artifacts associated with absolute single‐hemisphere measures and instead emphasizes the relationship between hemispheres for each individual. Moreover, the choice of accurate atlases is critical, as the parcellation used to delineate regions can lead to different interpretations. To date, Infant FreeSurfer (Zöllei et al. 2020) remains one of the most widely used pipelines to study brain development during infancy. Nevertheless, more recent efforts such as the Baby Open Brains project (Feczko et al. 2025) are producing more complete and comprehensive atlases, which may offer improved anatomical precision.

Finally, we should address the challenges of conducting research on heterogeneous populations. ASD is a neurodevelopmental disorder that arises from complex interactions between genes, brain circuitry, and environmental factors during early development (Hong et al. 2020). In addition, there is great variability in intellectual development, communication profiles, attentional functioning, trajectories, later outcomes, and more (Elsabbagh 2020). This clinical and etiological heterogeneity poses an obstacle for studies comparing ASD versus non‐ASD groups. Although these group comparisons, such as the one employed in this study, provide valuable insights, they often fail to capture the complex heterogeneity in ASD. Therefore, it is important to complement group‐level analyses with analytical approaches that capture this phenotypic diversity.

### 4.2 Future Directions

Future developmental studies should include other factors besides sex and risk that might impact brain development. Notably, socioeconomic factors have been associated with changes in EEG activity and brain structure (Brito et al. 2016; Noble et al. 2007). In addition, variability in diet, maternal education, prenatal exposure to toxic substances, and sleep patterns have been shown to contribute to individual differences in brain connectivity during infancy (Tomalski et al. 2013). Future studies should adopt a comprehensive approach that integrates these variables and their influence on brain development.

As previously noted, autism is a heterogenous condition, which encompasses many causes and endotypes. For this reason, we recommend that future studies include a more heterogeneous sample encompassing a wide range of symptoms and methods designed to tackle such heterogeneity. Recent advances in statistical models (Gartstein et al. 2020; Matusik et al. 2021) and transdiagnostic studies focused on children with shared neurobiological phenotypes (Kushki et al. 2021; Vandewouw et al. 2023) offer promising ways to tackle these challenges. Given the high heterogeneity, future studies should leverage dimensional or stratified models of ASD to advance our understanding of potential biomarkers.

## 5 Conclusion

Documenting the development of brain lateralization is a crucial first step toward improving our understanding of the etiology of ASD. Overall, our study showed that gamma band lateralization increases in infants who are later diagnosed with ASD. We also observed significant differences in the lateralization of the auditory network, an area thought to be important for language learning. The interplay between lateralization and developmental trajectories is complex, but measuring changes in infants can provide insights into factors contributing to ASD and its symptoms. More research is needed to understand how lateralization develops past infancy and how these differences can affect behavior, as this can help better inform clinical assessments and move toward more personalized treatments.

## Funding

This research was funded by FRQS‐Québec Autism Research Training (Blanco) as well as Brain Canada. The original data acquisition was funded by NIH P50 HD055782 (Webb) and the UK Medical Research Council (Basis Team—Johnson).

## Ethics Statement

The collection of the datasets employed in EEG‐IP underwent initial approval processes by the Ethics Review Boards of the participating institutions (Birkbeck University and The University of Washington).

## Consent

Informed consent was obtained from parents and/or guardians for each child that participated in this study according to each corresponding site, following the guidelines outlined in the Declaration of Helsinki. Following this, de‐identified data were transferred to EEG‐IP through formal data‐transfer agreements.

## Conflicts of Interest

The authors declare no conflicts of interest.

## Supporting information




**Figure S1** (A) Mean lateralization indices as a function of age (6–12 months) for each frequency band: theta, alpha, beta, and gamma. Contrasts between outcome groups: EL‐ASD− (purple), TL (coral), and EL‐ASD+ (yellow). Negative values represent right‐hemisphere dominance, whereas positive values represent left‐hemisphere dominance. (B) Mean lateralization indices across the EEG frequency spectrum. Cross‐sectional analyses for each time point: 6 months (top) and 12 months (bottom). The EL‐ASD+ group shows right hemisphere lateralization at 12 months of age, whereas the TL and EL‐ASD− groups show no lateralization. Confidence intervals were set at 90%, as represented by the shadowed areas. (C) Site differences in EEG frequency spectrum at 12 months of age. Analyses are presented for each of the testing sites: Seattle (left), London (middle), and both sites combined (right). (D) Changes in mean lateralization for each outcome group: TL (left), EL‐ASD− (center), and EL‐ASD+ (right). Analyses are presented for each frequency band: theta, alpha, beta, and gamma. All changes in lateralization between 6 and 12 months of age are significant, except for changes in gamma lateralization.
**Figure S2** Post hoc analyses between left hemisphere spectral power (gamma) at 6 and 12 months of age for ASD and non‐ASD groups.
**Figure S3** Distribution of lateralization values for all brain regions at 6 months (A) and 12 months (B) per outcome group.
**Table S1** Longitudinal breakdown of the sample and participant demographics.
**Table S2** Regions and their networks. Names of all brain regions.
**Table S3** Lateralization values for all bilateral regions at 6 and 12 months. These values only include infants who are typically developing.

## Data Availability

Given the restrictions regarding data‐sharing agreements between McGill University and each of the institutions involved in this project, the data used in this study are not publicly available. The datasets constituting EEG‐IP and analyzed during the current study are available from the lead authors of the original studies, upon reasonable request to basis@bbk.ac.uk for the London dataset and to sjwebb@uw.edu for the Seattle dataset. However, the code and analysis pipeline used to analyze the data are available at https://github.com/gabrielblancogomez/eegip_lateralization.
