# Supplementary material for: The Development of Lateralized Brain Oscillations in Infants: Lessons From Autism
Source: Dev Psychobiol. 2025 Nov 5;67(6):e70101. doi: 10.1002/dev.70101 (PMC12589938; doi:10.1002/dev.70101)
Supplement: Supplementary file 1 — Figure S1 (A) Mean lateralization indices as a function of age (6–12 months) for each frequency band: theta, alpha, beta, and gamma. Contrasts between outcome groups: EL‐ASD− (purple), TL (coral), and EL‐ASD+ (yellow). Negative values represent right‐hemisphere dominance, whereas positive values represent left‐hemisphere dominance. (B) Mean lateralization indices across the EEG frequency spectrum. Cross‐sectional analyses for each time point: 6 months (top) and 12 months (bottom). The EL‐ASD+ group shows right hemisphere lateralization at 12 months of age, whereas the TL and EL‐ASD− groups show no lateralization. Confidence intervals were set at 90%, as represented by the shadowed areas. (C) Site differences in EEG frequency spectrum at 12 months of age. Analyses are presented for each of the testing sites: Seattle (left), London (middle), and both sites combined (right). (D) Changes in mean lateralization for each outcome group: TL (left), EL‐ASD− (center), and EL‐ASD+ (right). Analyses are presented for each frequency band: theta, alpha, beta, and gamma. All changes in lateralization between 6 and 12 months of age are significant, except for changes in gamma lateralization. Figure S2 Post hoc analyses between left hemisphere spectral power (gamma) at 6 and 12 months of age for ASD and non‐ASD groups. Figure S3 Distribution of lateralization values for all brain regions at 6 months (A) and 12 months (B) per outcome group. Table S1 Longitudinal breakdown of the sample and participant demographics. Table S2 Regions and their networks. Names of all brain regions. Table S3 Lateralization values for all bilateral regions at 6 and 12 months. These values only include infants who are typically developing. [file DEV-67-e70101-s001.docx]

**APPENDIX**

**Participant demographics**

The original study consisted of EEG recordings from 195 infants, but only data from 175 participants was analyzed. A total of 117 participants had recordings at both time points. (Supplementary table 1). Of these, 58 had Elevated Likelihood (EL) for ASD (26 females, 28 from the London site) and 59 had typical likelihood (TL) for ASD (32 female, 27 from the London site).

**Supplementary Table 1 – Longitudinal Sample Breakdown**

*Participant Demographics by Likelihood Group*

| **ASD Likelihood** | **Participants at Both Timepoints** | **6-month Only** | **12-month Only** | **Total Unique Participants** |
| --- | --- | --- | --- | --- |
| EL | 58 | 11 | 21 | 90 |
| TL | 59 | 12 | 14 | 85 |
| **Total** | **117** | **23** | **35** | **175** |

**Group differences between EL-ASD and EL-No-ASD infants**

We ran a three-way repeated-measures ANOVA using age (6 months, 12 months) and frequency band (theta, alpha, beta, gamma) as within-subjects factors, and outcome (EL-No-ASD, TL, EL-ASD) as between-subject factors. Results can be observed in Supp. Figure 1.


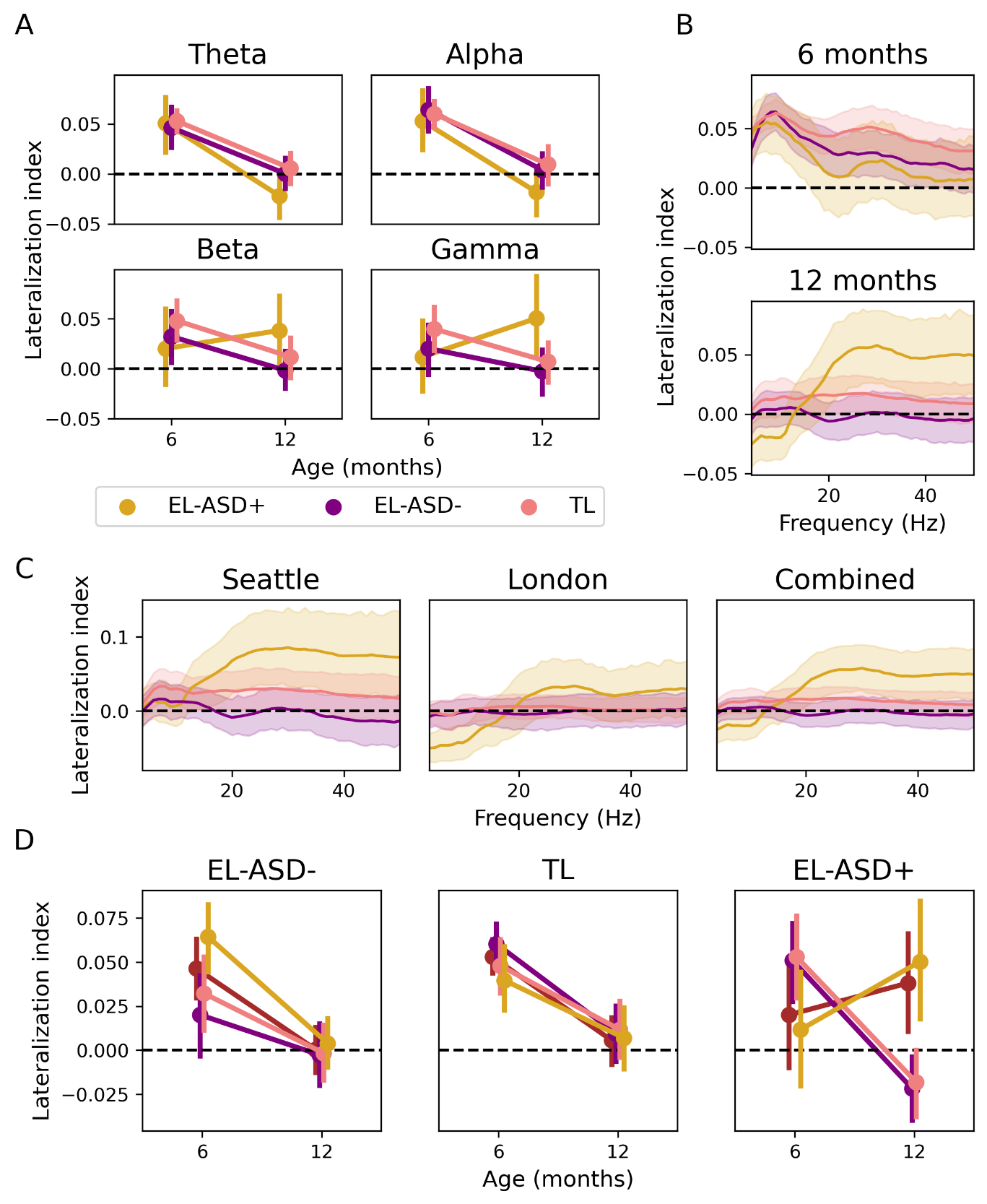


**Supp. Figure S1:**  **A)** Mean lateralization indices as a function of age (6 months to 12 months) for each frequency band: Theta, Alpha, Beta, and Gamma. Contrasts between outcome groups: EL-ASD- (purple), TL (coral), and EL-ASD+ (yellow). Negative values represent right-hemisphere dominance, while positive values represent left-hemisphere dominance. **B**) Mean lateralization indices across the EEG frequency spectrum. Cross-sectional analyses for each time point: 6 months (top) and 12 months (bottom). The EL-ASD+ group shows right hemisphere lateralization at 12 months of age, while the TL and EL-ASD- groups show no lateralization. Confidence intervals were set at 90%, as represented by the shadowed areas. **C**) Site differences in EEG frequency spectrum at 12 months of age. Analyses are presented for each of the testing sites: Seattle (left), London (middle) and both sites combined (right). **D**) Changes in mean lateralization for each outcome group: TL (left), EL-ASD- (center), and EL-ASD+ (right). Analyses are presented for each frequency band: Theta, Alpha, Beta, and Gamma. All changes in lateralization between 6 and 12 months of age are significant, except for changes in gamma lateralization.

**Differences by individual hemisphere**

Our findings show an overall decrease in lateralization from 6 months to 12 months across the whole brain. We conducted post-hoc tests to compare power differences for each individual hemisphere. Our analyses showed non-significant differences between ASD and no-ASD at 12 months for left hemisphere power (p=0.26) or right hemisphere power (p=0.47). Overall, these findings suggest that group differences are not driven by hemispheres alone but rather by their interaction. Additionally, we ran post-hoc tests to assess developmental changes to identify the source of the increased lateralization at 12 months in the ASD group. Our results showed that for infants in the ASD group, there was no significant change in power for each hemisphere between 6 and 12 months. We do, however, see a trend for the right hemisphere decreasing while the left hemisphere stays stable. On the other hand, for the no-ASD group, both the right and left hemispheres saw a significant decrease in lateralization from 6 to 12 months (p<0.01), perhaps suggesting that group differences are driven by a failure of ASD infants to undergo significant changes in lateralization. Nevertheless, we caution against comparing absolute power values. In this study, we focused primarily on a lateralization index, which is a “relative measure” or ratio defined as (left spectral power – right spectral power)/(left spectral power + right spectral power). Given the potential limitations of conducting source estimation, using a relative metric can reduce artifacts introduced by individual differences in skull thickness, electrode impedances, scalp conductivity, etc. In fact, relative measures of EEG power have been shown to have higher reproducibility and stability than absolute measures (Corsi-Cabrera et al., 2007; Duan et al., 2021).


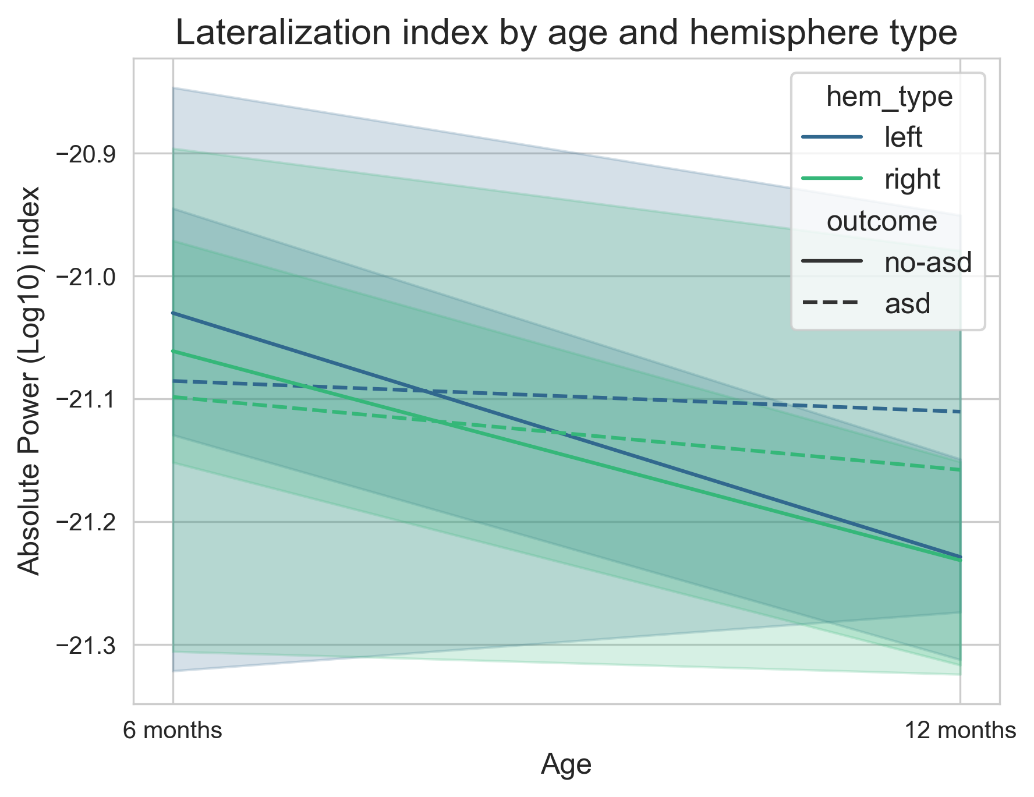


**Supp. Figure S2**: Post-hoc analyzes between left hemisphere spectral power (gamma) at 6 and 12 months of age for ASD and no-ASD groups.

**Selection of brain networks**

Resting-state networks can change significantly during development. In fact, studies multiple studies have shown that young children and infants do not always exhibit the same resting-state networks (Fransson et al., 2007; Wylie et al., 2014). Therefore, we decided to include resting state networks that have been found only in infants brains during the first year of life (Wang et al., 2021). In addition, we only selected three core resting-state networks that have been shown to be highly lateralized in previous studies, albeit in older children and adults (Agcaoglu et al., 2015). Finally, we also included the language network as defined by this study (Shirer et al., 2012) given that various studies suggest language networks develop early on during infancy (Tran et al., 2021). A list of all the regions that make up each network is found in Supplementary Table 2.

**Supplementary Table 2: Regions and their networks**

| **Network** | **Region** | **Source** |
| --- | --- | --- |
| **DMN** | Para hippocampal | (Wang et al., 2021) |
|  | Isthmus Cingulate |  |
|  | Posterior Cingulate |  |
|  | Precuneus |  |
|  | Inferior Parietal |  |
| **Auditory** | Superior Temporal |  |
|  | Middle Temporal |  |
|  | Insula |  |
| **Visual** | Cuneus |  |
|  | Pericalcarine |  |
|  | Lingual |  |
|  | Lateral Occipital |  |
| **Language** | Pars Opercularis | (Shirer et al., 2012) |
|  | Pars Orbitalis |  |
|  | Pars Triangularis |  |
|  | Middle Temporal |  |
|  | Superior Temporal |  |
|  | Supramarginal |  |
|  | Inferior Parietal |  |

**Distribution of lateralization values for all regions**


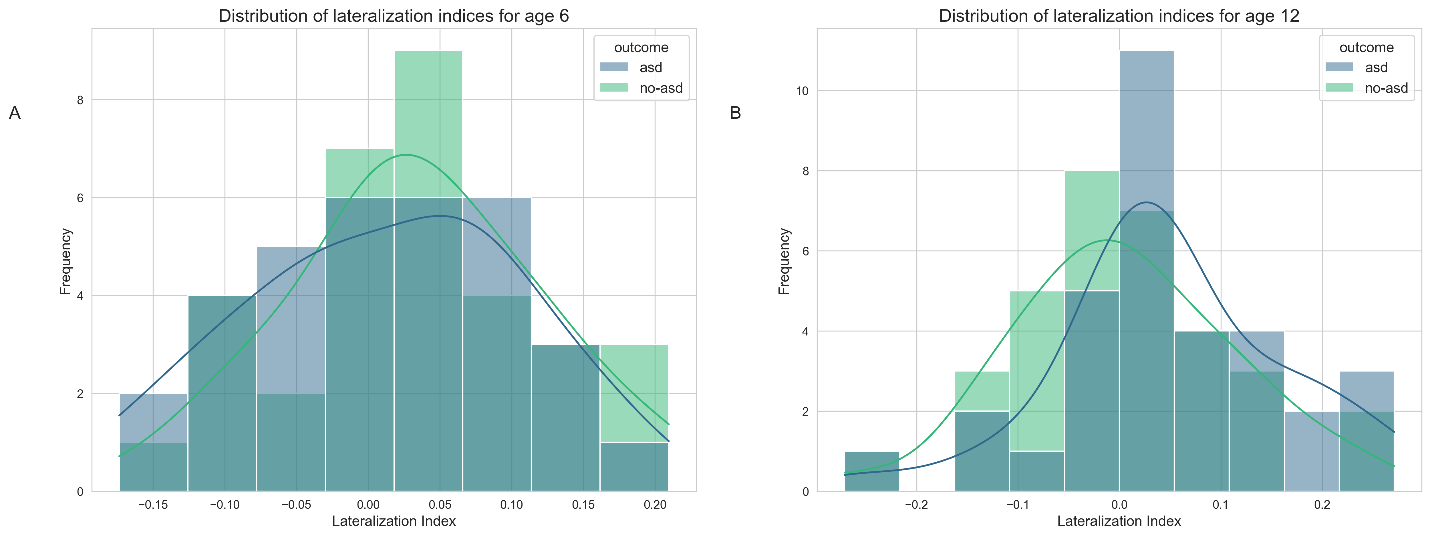


**Supp. Figure S3:** Distribution of lateralization values for all brain regions at 6 months (**A**) and 12 months (**B**) per outcome group.

**Supplementary Table 3:** Lateralization values for all bilateral regions at 6 and 12 months. These values only include infants who are typically developing.

| Name | Lateralization Index | Absolute Lateralization Index | Age |
| --- | --- | --- | --- |
| caudalmiddlefrontal | 0.23 | 0.23 | 6 |
| superiortemporal | 0.22 | 0.22 | 6 |
| supramarginal | 0.2 | 0.2 | 6 |
| middletemporal | 0.15 | 0.15 | 6 |
| parahippocampal | -0.13 | 0.13 | 6 |
| rostralmiddlefrontal | 0.12 | 0.12 | 6 |
| bankssts | 0.11 | 0.11 | 6 |
| temporalpole | -0.1 | 0.1 | 6 |
| parsorbitalis | 0.1 | 0.1 | 6 |
| medialorbitofrontal | 0.1 | 0.1 | 6 |
| isthmuscingulate | 0.08 | 0.08 | 6 |
| rostralanteriorcingulate | -0.08 | 0.08 | 6 |
| fusiform | -0.08 | 0.08 | 6 |
| superiorparietal | 0.06 | 0.06 | 6 |
| inferiorparietal | 0.06 | 0.06 | 6 |
| precuneus | 0.05 | 0.05 | 6 |
| insula | 0.05 | 0.05 | 6 |
| paracentral | 0.05 | 0.05 | 6 |
| posteriorcingulate | 0.05 | 0.05 | 6 |
| postcentral | 0.05 | 0.05 | 6 |
| lingual | -0.04 | 0.04 | 6 |
| caudalanteriorcingulate | 0.03 | 0.03 | 6 |
| lateraloccipital | 0.03 | 0.03 | 6 |
| parsopercularis | -0.02 | 0.02 | 6 |
| pericalcarine | 0.02 | 0.02 | 6 |
| inferiortemporal | -0.02 | 0.02 | 6 |
| lateralorbitofrontal | 0.01 | 0.01 | 6 |
| frontalpole | 0.01 | 0.01 | 6 |
| superiorfrontal | -0.01 | 0.01 | 6 |
| parstriangularis | -0.01 | 0.01 | 6 |
| precentral | 0 | 0 | 6 |
| cuneus | 0 | 0 | 6 |
| transversetemporal | 0 | 0 | 6 |
| medialorbitofrontal | -0.28 | 0.28 | 12 |
| parsorbitalis | 0.23 | 0.23 | 12 |
| inferiorparietal | 0.22 | 0.22 | 12 |
| cuneus | 0.16 | 0.16 | 12 |
| caudalmiddlefrontal | 0.16 | 0.16 | 12 |
| insula | -0.14 | 0.14 | 12 |
| pericalcarine | -0.13 | 0.13 | 12 |
| parsopercularis | -0.13 | 0.13 | 12 |
| superiortemporal | 0.12 | 0.12 | 12 |
| isthmuscingulate | 0.12 | 0.12 | 12 |
| parahippocampal | -0.12 | 0.12 | 12 |
| inferiortemporal | 0.11 | 0.11 | 12 |
| precuneus | 0.11 | 0.11 | 12 |
| supramarginal | -0.09 | 0.09 | 12 |
| rostralanteriorcingulate | -0.08 | 0.08 | 12 |
| paracentral | 0.06 | 0.06 | 12 |
| postcentral | 0.06 | 0.06 | 12 |
| transversetemporal | -0.05 | 0.05 | 12 |
| lateralorbitofrontal | 0.04 | 0.04 | 12 |
| parstriangularis | -0.04 | 0.04 | 12 |
| lateraloccipital | -0.03 | 0.03 | 12 |
| middletemporal | -0.03 | 0.03 | 12 |
| frontalpole | 0.03 | 0.03 | 12 |
| caudalanteriorcingulate | -0.03 | 0.03 | 12 |
| posteriorcingulate | 0.03 | 0.03 | 12 |
| lingual | 0.02 | 0.02 | 12 |
| temporalpole | -0.02 | 0.02 | 12 |
| superiorparietal | 0.02 | 0.02 | 12 |
| precentral | 0.01 | 0.01 | 12 |
| rostralmiddlefrontal | -0.01 | 0.01 | 12 |
| fusiform | 0.01 | 0.01 | 12 |
| bankssts | -0.01 | 0.01 | 12 |
| superiorfrontal | 0 | 0 | 12 |
